# Validation of the GenoType^® ^MTBDRplus assay for diagnosis of multidrug resistant tuberculosis in South Vietnam

**DOI:** 10.1186/1471-2334-10-149

**Published:** 2010-06-03

**Authors:** Mai NT Huyen, Edine W Tiemersma, Nguyen TN Lan, Frank GJ Cobelens, Nguyen H Dung, Dinh N Sy, Tran N Buu, Kristin Kremer, Pham T Hang, Maxine Caws, Richard O'Brien, Dick van Soolingen

**Affiliations:** 1 Phạm Ngọc Thạch hospital, 120 Hung Vuong, district 5, Ho Chi Minh City, Viet Nam; 2 KNCV Tuberculosis Foundation, PO Box 146, 2501 CC The Hague, The Netherlands; 3 Center for Poverty-related Communicable Diseases, Amsterdam Institute of Global Health and development, Academic Medical Center, Meibergdreef 9, 1105 AZ Amsterdam, The Netherlands; 4 National Lung Hospital, 463 Hoang Hoa Tham, Ba Dinh district, Ha Noi, Viet Nam; 5 Tuberculosis Reference Laboratory, RIVM, PO Box 1, 3720 BA Bilthoven, The Netherlands; 6 Wellcome Trust Major Overseas Programme, Oxford University Clinical Research Unit, Hospital for Tropical Diseases,190 Ben Ham Tu, District 5, Ho Chi Minh City, Viet Nam; 7 Foundation for Innovative New Diagnostics (FIND), Avenue de Budé 16, 1202 Geneva, Switserland; 8 Departments of Pulmonary Diseases and Medical Microbiology, Radboud University, PO box 9101, 6500 HB Nijmegen, The Netherlands

## Abstract

**Background:**

To control multidrug resistant tuberculosis (MDR-TB), the drug susceptibility profile is needed to guide therapy. Classical drug susceptibility testing (DST) may take up to 2 to 4 months. The GenoType^® ^MTBDR*plus *test is a commercially available line-probe assay that rapidly detects *Mycobacterium tuberculosis *(MTB) complex, as well as the most common mutations associated with rifampin and isoniazid resistance.

We assessed sensitivity and specificity of the assay by using a geographically representative set of MTB isolates from the South of Vietnam.

**Methods:**

We re-cultured 111 MTB isolates that were MDR, rifampin-resistant or pan-susceptible according to conventional DST and tested these with the GenoType^® ^MTBDR*plus *test.

**Results:**

By conventional DST, 55 strains were classified as MDR-TB, four strains were rifampicin mono-resistant and 52 strains were susceptible to all first-line drugs. The sensitivity of the GenoType^® ^MTBDR*plus *was 93.1% for rifampicin, 92.6% for isoniazid and 88.9% for the combination of both; its specificity was 100%. The positive predictive value of the GenoType^® ^MTBDR*plus *test for MDR-TB was 100% and the negative predictive value 90.3%.

**Conclusions:**

We found a high specificity and positive predictive value of the GenoType^® ^MTBDR*plus *test for MDR-TB which merits its use in the MDR-TB treatment program in Vietnam.

## Background

Tuberculosis (TB) is an important disease in developing countries [1]. A major concern is the occurrence of multidrug resistant (MDR) TB [2] which is characterized by resistance to both rifampicin and isoniazid (INH). MDR-TB is difficult to treat and associated with increased treatment failures and relapses. In patients with previously untreated pulmonary TB, inappropriate treatment may lead to selection for bacteria with drug resistance associated mutations [3].

Vietnam ranks 14^th ^among the countries with the highest burden of TB; the incidence of TB is highest in the southern part of the country [1]. In 2005, South Vietnam notified 29,789 new smear positive cases yielding an estimated prevalence of 92.8 per 100,000 (NTP, unpublished data, 2006). *M. tuberculosis *resistance to INH is common (16-25% among new patients) [4,5]. Among patients experiencing a first episode of TB these two studies reported MDR-TB rates of 2 and 4%, and of 23% and 27% among previously treated patients, respectively [4,5], whereas 80% chronic patients had MDR-TB [6]. Genetically, approximately half of the strains belongs to the East-African Indian clade whereas the other half are of the Beijing genotype, which was found to be strongly associated with (multi-)drug resistance [7,8].

To control MDR-TB, drug resistance patterns should be available to guide the therapy of the patient. However, phenotypic drug susceptibility testing (DST) is a time-consuming process because it requires culturing, which may take up to two months or longer. As long as no DST results are available, the patient will be treated with standard first-line anti-TB drugs. Rapid diagnosis of MDR-TB will permit an earlier start with second-line drug treatment for patients with MDR-TB and may thus decrease the risk of treatment failure, relapse, amplification of DR, and continuing transmission of MDR-TB.

The vast majority of resistance to rifampicin is caused by mutations located in the 81-bp hotspot region of the *rpoB *gene [9]. Mutations conferring resistance to INH are located at several genomic loci (*katG, inhA *and *kasA*) [10-14]. Varying by geographic area, 50 to 100% of INH resistant strains has mutations in codon 315 of the *katG *gene or in the promoter region of the *inhA *gene [13,15,16].

The GenoType^® ^MTBDR*plus *assay (Hain Lifescience, Nehren, Germany) is a commercially available assay that combines detection of MTB complex with prediction of resistance to rifampicin and INH. In the assay a multiplex PCR is followed by hybridization of the obtained DNA amplicons to membrane-bound probes. The assay combines detection of MTB complex with detection of mutations in the 81-bp hotspot region of *rpoB*, at codon 315 of the *katG *gene and in the *inhA *promoter region. It was found to have high sensitivity and high specificity for rifampicin and INH resistance and performs well when applied directly to AFB smear-positive sputum specimens [15,17-20]. A recent meta-analysis pooled all these studies and calculated pooled sensitivity and specificity rates of 99% (95% confidence interval (CI), 96%-100%) and 99% (95% CI, 98%-100%) respectively for rifampicin resistance, and of 96% (93-98%) and 100% (99-100%), respectively for isoniazid resistance [21].

In June 2008, the World Health Organization (WHO) endorsed the use of molecular line-probe assays for MDR-TB screening [22], and the GenoType^® ^MTBDR*plus *assay has since been introduced for routine practice in various countries [15,17-20,23]. The WHO recommends that before using the assay in routine TB treatment and control, the performance of the assay in relation to the locally circulating *M. tuberculosis *bacteria should be validated [22].

The National Tuberculosis Control Program of Vietnam intends to use this test in support of Programmatic Management of DR-TB (PMDT) on sputum specimens of all MDR-TB suspects (i.e., those failing category 1 treatment and those being smear-positive after 3 months of category 2 treatment). The Genotype^® ^MTBDRplus assay will be used to select patients with rifampicin resistant isolates for PMDT. Therefore, we assessed the test's sensitivity and specificity in diagnosing MDR-TB at the laboratory of Pham Ngoc Thach Hospital (PNTH) using a geographically representative set of *M. tuberculosis *isolates with known phenotypic resistance patterns from the South of Vietnam.

## Methods

### Study population

We selected MTB isolates from the latest nationwide TB drug resistance survey (DRS) in Vietnam conducted in 2004-2005. This survey was carefully designed as to cover all geographical parts of Vietnam and the set of samples thus provided a national estimate of drug resistance for Vietnam. DST was done by the two national reference laboratories, one of which was at PNTH in Ho Chi Minh City. The DRS was part of the WHO/IUATLD Global Project on TB Drug Resistance Surveillance and followed WHO guidelines [24]. PNTH's laboratory participates in an annual international proficiency study on DST together with laboratories in Korea and Australia, with results that are concordant to those of other laboratories for more than 95% of the tests.

Complete DST results were available for 1,826 patients with smear-positive TB from 80 different TB clinics throughout the country. Of these, 1,044 (57%) specimens were collected in the South of Vietnam and tested in PNTH (910 from new patients and 134 from previously treated patients). Sputum specimens were processed according to standard procedures [24]. After centrifugation, sediment was inoculated on Löwenstein-Jensen (LJ) medium and incubated at 37°C for 4 to 8 weeks followed by species identification of culture-positive samples. DST for isoniazid (0.2 μg/ml), streptomycin (4 μg/ml), ethambutol (2 μg/ml), and rifampicin (40 μg/ml) was performed using LJ media following the proportion method [25]. Isolates from all 30 new and 29 re-treatment cases that were either identified as MDR-TB (n = 55) or resistant to rifampicin (n = 4) by phenotypic DST were included in this study. In addition, from the isolates that were susceptible to all tested first-line drugs from new and re-treatment patients, we randomly selected 52 isolates, so that the total number of tested strains was 111.

### GenoType^® ^MTBDR*plus *testing

GenoType^® ^MTBDR*plus *testing was performed blinded from the phenotypic DST results. The selected MTB isolates were re-cultured from the -70°C freezer and subjected to the GenoType^® ^MTBDR*plus *test according to the manufacturer's recommendations http://www.hain-lifescience.de. By multiplex PCR the *rpoB*, *katG *and *inhA *genes were amplified and the resulting biotin-labeled amplicons were hybridized to DNA probes bound to membrane strips. Hybridization was detected by addition of a streptavidin/alkaline phosphatase (AP) conjugate and an AP mediated staining reaction. For each gene, the GenoType^® ^MTBDR*plus *assay tests for presence of so-called wild-type (WT) and mutant (MUT) probes, the first comprising the most important resistant areas of the respective genes and the second some of the most common resistance mediating mutations. Next to that, the TUB zone hybridizes with amplicons generated from all members of the Mycobacterium complex and can thus serve for species identification. The membrane-bound DNA probes included eight *rpoB *wild-type probes, four *rpoB *mutant probes (with D516V, H526Y, H526D, and S531L mutations), one *katG *wild-type probe, two *katG *mutant probes (with S315T1 and S315T2 mutations), two *inhA *wild-type probes and four *inhA *mutant probes (with C15T, A16G, T8C, and T8A mutations) [26]. Following the manufacturer's instructions http://www.hain-lifescience.de, susceptibility to isoniazid and rifampicin was defined as hybridization to all the wild type probes and no hybridization to the mutant probes. A strain that revealed hybridization to both a mutant probe and to the corresponding wild type probe was considered to represent a heterogeneous population of bacteria or a mixed infection of a sensitive and a resistant strain.

### Species identification

*M. tuberculosis *was identified by smear microscopy followed by a positive niacine reaction [27]. Further species identification was performed using Innolipa Mycobacteria v2 (Innogenetics, Gent, Belgium) if a discrepancy was found between initial species identification and results from the GenoType^® ^MTBDRplus assay (i.e., no hybridization with the TUB band).

### DNA fingerprinting and sequencing

In order to identify possible cases of mixed infection, spoligotyping [28] and IS*6110 *restriction fragment length polymorphism (RFLP) [29] were applied to single colonies growing on standard culture medium and medium supplemented with tuberculostatics. DNA patterns were scanned and analysed by using Gelcompar software (Applied Maths, Sint-Martens-Latum, Belgium) as previously described [30]. The Beijing genotype was defined by spoligotyping as any isolate without Direct Repeat spacers 1-34 and the presence of ≥3 of the spacers 35-43 [31]. Other genotypes were defined as described by Brudey *et al*. [32], including the Vietnam genotype (EAI-VNM) that belongs to the East African Indian genotype family of *M. tuberculosis *and is the most frequent genotype in this study site [8].

Sequencing of the rifampin resistance-determining region (RRDR) region of the *rpoB *gene was performed for strains that had discordant results for rifampicin according to conventional DST and the GenoType^® ^MTBDR*plus *test. The 350 bp fragment of the *rpoB *gene was amplified using outer primers RPOBF (5'-GGGAGCGGATGACCACCCA3') and RPOBR (5'-GCGGTACGGCGTTTCGATGAAC-3'). The primers were designed using Primer Express version 2.0 software (Applied Biosystems Inc, Foster City, CA, USA) [33].

### Ethical approval

The study protocol was approved by the Research Board of Pham Ngoc Thach hospital. Since this study used stored isolates with known drug resistance patterns and no additional procedures on the patients were involved, individual informed consent was not obtained.

### Statistical Methods

Data were double entered in EpiData version 3.1 http://www.epidata.dk by two separate study assistants. Discrepancies were checked against the crude data. Chi-squared tests were performed using Epi Info version 6.04 http://www.cdc.gov/epiinfo/. Results were considered significant at P < 0.05.

## Results

### Concordance between conventional DST and GenoType^® ^MTBDR*plus *assay

Of 111 isolates tested, there was one MDR strain that lacked the TUB band. Although this strain was earlier identified as *M. tuberculosis*, further species identification identified the strain as *M. avium-intracellulare *(MAIS). Spoligotyping after re-culturing this isolate showed that it was a mixture of *M. tuberculosis *and MAIS. Since the GenoType^® ^MTBDR*plus *assay did not identify this strain as MDR-TB, all analyses describing the assay's performance include 110 isolates, of which 58 were resistant to rifampicin by conventional DST.

Based on phenotypic DST, 54 strains were MDR-TB, four strains were rifampicin mono-resistant and 52 strains were susceptible to all first-line drugs. Considering the phenotypic DST method as the gold standard, the GenoType^® ^MTBDR*plus *test correctly identified 48 of 54 MDR-TB strains (88.9%, 95% CI: 77.4-95.8%); 54 of 58 rifampicin resistant strains (93.1%, 95% CI: 83.3-98.1%); 50 of 54 INH resistant strains (92.6%, 95% CI: 82.1-97.9%); and all susceptible strains (100%, 95% CI: 93.2-100%). The specificity for detecting MDR-TB was 100%. The overall concordance of the GenoType^® ^MTBDR*plus *test and phenotypic DST was 94.5% (104/110). Sensitivities, specificities and predictive values are listed in Table 1.

**Table 1:** The sensitivity, specificity, positive predictive value (PPV) and negative predictive value (NPV) and 95% confidence intervals of the GenoType^® ^MTBDR*plus *assay on 111 *M. tuberculosis *cultures isolates in the South of Vietnam.

	Rifampicin	Isoniazid	Multi drug resistance
**Sensitivity**	93.1% (83.3-98.1)	92.6% (82.1-97.9)	88.9% (77.4-95.8)
**Specificity**	100% (93.2-100)	100% (93.6-100)	100% (93.6-100)
**PPV**	100% (93.4-100)	100% (92.9-100)	100% (92.6-100)
**NPV**	92.9% (82.7-98.0)	93.3% (83.8-98.1)	90.3% (80.1-96.4)

Sequencing the RRDR region of the *rpoB *gene of the four strains with discordant results for rifampicin revealed that one strain possessed the H526L mutation which is not present on the membrane strips; in the three remaining strains no mutations were detected in the *rpoB *gene.

### Frequency of drug resistance associated mutations

Mutation patterns produced by the GenoType^® ^MTBDR*plus *test are displayed in Table 2. Among the 54 RIF resistant TB strains detected by this test, the frequency of *rpoB *mutations was: 27 S531L (50%), 6 H526Y (10.9%), 3 D516V (5.5%), 1 H526D (1.8%), 7 missing WT2 (12.7%), 10 missing WT3 (18.1%), 6 missing WT4 (10.9%), 14 missing WT7 (25.5%), 26 missing WT8 (48%), and no case missing WT1, WT5 or WT6.

**Table 2:** Mutation patterns following from the GenoType ^® ^MTBDR*plus *assay.

*rpoB *mutations	*katG *mutations	*inhA *mutations	Frequency	Proportion
D516V	S315T1	--	1	0.9
D516V	unknown *	--	1	0.9
D516V	--	C15T	1	0.9
H526D	S315T1	--	1	0.9
H526Y	S315T1	--	5	4.6
S531L	unknown *	--	2	1.8
S531L	S315T1	C15T	1	0.9
S531L	S315T1	--	16	14.6
S531L	S315T2	--	1	0.9
S531L	--	C15T	4	3.6
S531L	--	--	2	1.8
H526Y + S531L	--	--	1	0.9
unknown *	S315T1	--	13	11.8
unknown *	--	--	3	1.8
unknown *	unknown *	C15T	1	0.9
unknown *	--	C15T	1	0.9
--	S315T1	--	1	0.9
--	--	C15T	1	0.9

**Total number of strains with any mutations**			**56**	**50.9**

--	--	--	54	49.1

**Total number of strains**			**110**	**100**

* unknown mutation: no hybridization to one or more of the wild type probes nor to any of the mutation probes.

Among 50 INH resistant TB strains as identified by the GenoType^® ^MTBDRplus test, *katG *mutations occurred in 43 (86%) and *inhA *mutations in 9 strains (18%). Two of the 43 (5%) strains with a *katG *codon 315 mutation had an additional mutation in the *inhA *promoter region. The most frequently observed *katG *mutation was *katG *S315T1 (in 38 of 43 strains, 88.4%), whereas *katG *S315T2 (2.3%) and unknown mutations (i.e., no hybridization to the *katG *WT nor to either of the mutation probes, 9.3%) occurred less frequently. All 9 strains with a mutation in the *inhA *promoter region had an *inhA *C15T mutation (Table 2).

### Detection of mixed bacterial populations

**Figure 1:**
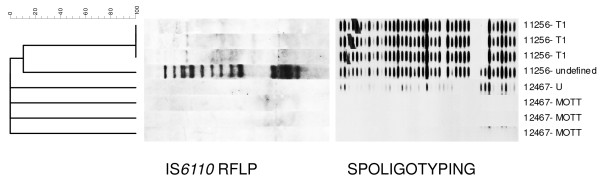
**Patterns of multiple *Mycobacterium spp*. infections as obtained by RFLP and spoligotyping techniques**. The first three isolates have only one band by RFLP (non-Beijing) and spoligotype T1; the 4^th ^sample is a mixture of Beijing genotype (as obtained by RFLP) and spoligotype T1. The 5^th ^sample has no RFLP banding pattern and an undefined spoligotype, whereas for the last three isolates no banding patterns were found by RFLP and spoligotyping, probably as a result of the presence of non-tuberculous mycobacteria (NTM).

With this rapid assay four possible mixtures were detected, although one of these was not identified by the assay as *M. tuberculosis *due to a lacking TUB band and was later identified to contain MAIS. These four phenotypically rifampicin resistant isolates were demonstrated to carry mutations in the *rpoB *gene and/or in the *katG *gene or the *inhA *promoter region, but did not lack hybridization on any of the wild type probes. By using DNA fingerprinting one of these isolates was confirmed to be a mixture of two MTB strains (spoligotype T1 and an undefined type; RFLP type T1 and Beijing), and the isolate lacking the TUB band (number 12647) was identified as a mixture of a MTB strain (spoligotype U) and a non-tuberculous mycobacterium (note that spoligotyping yielded a weak signal for *M. tuberculosis *for one single colony culture of this isolate) (Table 3, Figure 1). In the two remaining samples mixed bacterial populations could not be detected. After spoligotyping which revealed no differences as it has a very low resolution among Beijing strains, we also performed IS*6110 *RFLP typing on single colony cultures of each of these two samples and found they all had identical banding patterns (Table 3).

**Table 3:** Identification of mixed TB infections by spoligotyping and RFLP.*

Strain no.	TUB	Rifampin		Isoniazid		Spoligotype	RFLP type + spoligotype
		WT	MUT	WT	MUT		
10484	+	+	Mut 2A & 3	+	-	Beijing	Identical Beijing
11256	+	+	Mut 3	+	*katG *Mut 1 &*inhA *Mut 1	T1 + undefined type	T1 +BEIJING
11901	+	+	Mut 3	+	*inhA *Mut 1	Beijing	Identical Beijing
12467	-	-	+	+	*katG Mut 1*	U + No band	U + no band

*TUB: *M. tuberculosis *confirmation band; WT: wild type; MUT: mutant

## Discussion

In this study, the GenoType^® ^MTBDR*plus *assay correctly identified 93.1%, 92.6% and 88.9% of the rifampicin, INH and combined resistance (MDR), respectively and had 100% specificity for each. The sensitivity of detection of rifampicin resistance was similar to that reported from Germany, Italy, Finland, France, Denmark, Turkey and Taiwan (92-100%, p > 0.05) [15,17-20,26,34,35]. The GenoType^® ^MTBDR*plus *assay failed to detect four (6.8%) of the rifampicin resistant strains in our study, which was caused either by a rare mutation which is not present on the strips (n = 1) or by probable mutations in other genomic regions of the *rpoB *gene (n = 3). The sensitivity for detection of isoniazid resistance in our study was 91.2%, which was similar to reports from Germany, Finland, Denmark and Taiwan (84 - 100%, p > 0.05) [15,17-20,26,34,35], but higher than reported from Turkey, Italy, France and the Caribbean (35-73%, P < 0.05) [17,18,20,36]. For MDR-TB the sensitivity of the test was somewhat lower than reported from other research [21].

The distribution of mutations identified in our study is significantly different from that reported from other continents. We found that the S531L mutation in the *rpoB *gene occurred most frequently (50%) among rifampicin resistant strains. This mutation occurred even more frequently in a collection of isolates from South Africa (70.5%, p = 0.014) [23]. In our study only 36 (66.7%) MDR-TB strains had a mutation that could be identified by one of the four *rpoB *mutant probes present on the strip, whereas this was much higher in South Africa (91% (81/89)) [23]. Thus, the assay performs better detecting *rpoB *specific mutations that confer rifampicin resistance in South Africa than in Vietnam. For INH resistance, mutations in the *katG *gene were by far most common (86%) and the S315T1 mutation was found most frequently (88.4%) in our population. From South Africa, Van Rie et al [37] reported similar results, whereas Barnard and colleagues [23] reported that this mutation occurred less frequently (37.6%; p = 0.01). In our study 18% of INH resistant MTB strains carried a mutation in the *inhA *promoter region (invariably C15T) which is considerably lower than the 40% reported in Barnard's study (p = 0.007). It should be noted that we only tested MDR-TB isolates, whereas Barnard's study also included INH monoresistant strains and MDR-TB may be primarily associated with *katG *mutations that generally confer high levels of INH resistance [38].

The GenoType^® ^MTBDR*plus *assay can detect mixtures of drug resistant and drug susceptible bacterial populations. In this study four mixtures were found: three mixtures of *M. tuberculosis *strains and one mixture of a *M. tuberculosis *strain and an *M. avium *complex strain. This indicates that even in a high prevalence area like Vietnam a minor proportion of the TB cases is caused by a mixture of non-tuberculous mycobacteria and MTB. The GenoType^® ^MTBDR*plus *assay may not be sensitive enough to be used for species identification in case of mixed bacterial populations, since the *M. tuberculosis *and *M. avium *mixture revealed no TUB band.

Mixed infections were confirmed by typing a limited number of single colony cultures by spoligotyping and RFLP. In two samples that seemed to consist of a mixture when tested with the GenoType^® ^MTBDR*plus *assay, presence of a mixed infection could not be confirmed which could have been the result of testing only a limited number of single colony cultures.

With the 100% specificity of the GenoType^® ^MTBDR*plus *assay to detect MDR in *M. tuberculosis *isolates, no patient would be inappropriately treated with category 4 (MDR-TB) treatment if this test would be used in routine for rapid MDR-TB diagnosis. On the other hand, 6.9% of patients would not receive appropriate category 4 treatment (which is based on detection of rifampicin resistance) if identification of MDR-TB patients is done using only the GenoType^® ^MTBDR*plus *test.

## Conclusions

Overall, the GenoType^® ^MTBDR*plus *test is reliable, rapid and easy to perform for the simultaneous detection of rifampicin and INH resistance in *M. tuberculosis*. With high sensitivity for detection of rifampin resistance and high specificity for MDR, we conclude that this test strongly facilitates adequate treatment of MDR-TB patients, long before the results of conventional DST are available. Because discordance still exists between the conventional and molecular approach of DST and susceptibility of bacteria to drugs is defined as inhibition of growth, we recommend that the GenoType^® ^MTBDR*plus *test should serve as an early guidance of therapy, which should be followed by a phenotypic DST confirmation for all suspected MDR-TB patients. Incorporation of the molecular test in the National Tuberculosis Program is an important step forward in the rapid diagnosis of MDR-TB among suspected patients in the PMDT program. The application of the molecular test directly to clinical material with sufficient bacteria will further speed up the turnaround time of the rapid diagnosis of MDR-TB and will be the next step of implementation.

## List of abbreviations

AFB: acid-fast bacilli; AP: alkaline phosphatase; CI: confidence interval; DRS: drug resistance survey; DST: drug susceptibility testing; FIND: Foundation for Innovative New Diagnostics; INH: isoniazid; IUATLD: International Union Against Tuberculosis and Lung Disease; LJ: Löwenstein-Jensen; MAIS: *Mycobacterium avium-intracellulare*; MDR-TB: multidrug resistant tuberculosis; MTB: *Mycobacterium tuberculosis*; NPV: negative predictive value; PMDT: Programmatic Management of drug resistant TB; PNTH: Pham Ngoc Thach Hospital; PPV: positive predictive value; TB: tuberculosis; RFLP: restriction fragment length polymorphism; RRDR: rifampin resistance-determining region; WHO: the World Health Organization.

## Competing interests

The authors declare that they have no competing interests.

## Authors' contributions

MH was involved in data collection and analysis and drafted the manuscript. ET and FC were involved in the conception, design and coordination of the study. NL was involved in the conception of the study and coordinated the study including collection of the strains. DS, ND, and TB were involved in conception of the study and in coordinating the collection of the strains used in this study. KK and DS gave guidance to the analysis and interpretation of the molecular results. PH did the drug susceptibility tests for this study. RO was involved in the conception and design of the study. MC supervised the sequencing and strain identification. All authors read and approved the final manuscript.

## Pre-publication history

The pre-publication history for this paper can be accessed here:

http://www.biomedcentral.com/1471-2334/10/149/prepub
